# The CLC-5 2Cl^−^/H^+^ exchange transporter in endosomal function and Dent's disease

**DOI:** 10.3389/fphys.2012.00449

**Published:** 2012-11-30

**Authors:** Jonathan D. Lippiat, Andrew J. Smith

**Affiliations:** ^1^School of Biomedical Sciences, Faculty of Biological Sciences, University of LeedsLeeds, UK; ^2^Faculty of Life Sciences, University of ManchesterManchester, UK

**Keywords:** CLC-5, Dent's disease, endocytosis, endosomal acidification, chloride transport

## Abstract

CLC-5 plays a critical role in the process of endocytosis in the proximal tubule of the kidney and mutations that alter protein function are the cause of Dent's I disease. In this X-linked disorder impaired reabsorption results in the wasting of calcium and low molecular weight protein to the urine, kidney stones, and progressive renal failure. Several different ion-transporting and protein clustering roles have been proposed as the physiological function of CLC-5 in endosomal membranes. At the time of its discovery, nearly 20 years ago, it was understandably assumed to be a chloride channel similar to known members of the CLC family, such as CLC-1, suggesting that chloride transport by CLC-5 was critical for endosomal function. Since then CLC-5 was found instead to be a 2Cl^−^/H^+^ exchange transporter with voltage-dependent activity. Recent studies have determined that it is this coupled exchange of protons for chloride, and not just chloride transport, which is critical for endosomal and kidney function. This review discusses the recent ideas that describe how CLC-5 might function in endosomal membranes, the aspects that we still do not understand, and where controversies remain.

## Introduction

Like several other proteins that mediate the movement of Cl^−^ ions across membranes, CLC-5 was identified as a protein defective in human disease. In this case, the search for genes underlying Dent's disease, an inherited kidney disorder, identified the *CLCN5* gene and sequence variations in Dent's patients (Fisher et al., 1995; Lloyd et al., 1996). In this X-linked disorder, individuals have severely reduced kidney function with defective reabsorption of low molecular weight protein (e.g., albumin, hormone and vitamin binding proteins, microgobulins) and Ca^2+^ by the renal tubules, which appear at unusually high levels in urine. Kidney stones are frequently observed in Dent's patients, occasionally rickets, and the disorder often progresses to complete renal failure. Many different mutations have been identified, including nonsense, missense, and mis-splicing, and which affect protein function, processing, or trafficking (Grand et al., 2009; Smith et al., 2009; Lourdel et al., 2012).

CLC-5 is a member of the CLC family of Cl^−^ channels and 2Cl^−^/H^+^ transporters, still known today as the voltage-gated chloride channel family, and have 9 human members. This classification was based on the functional properties of CLC-1, a plasma membrane Cl^−^ channel that is involved in skeletal muscle excitability. CLC-1 exhibits outward rectification through increased open probability when the membrane becomes depolarized (Jentsch et al., 1995). These properties, however, proved to be the exception rather than the defining characteristics of the whole protein family: CLC-2 is inwardly-rectifying, CLC-Ka and Kb exhibit little voltage-dependence and generally form a Cl^−^ leak conductance, whilst the other members CLC–3 to 7 have more recently been shown to function as outwardly-rectifying 2Cl^−^/H^+^-exchange transporters (Picollo and Pusch, 2005; Scheel et al., 2005; Neagoe et al., 2010; Leisle et al., 2011) and are predominantly located in the membranes of intracellular organelles, particularly endosomes, lysosomes, and exocytic vesicles.

Early studies led to the proposal that CLC-5 played a supporting role to H^+^-ATPase, which actively pumps H^+^ into endosomes, by providing a Cl^−^-permeable shunt conductance. This process maintains endosomal acidification because passive entry of Cl^−^ into endosomes, flowing down an electrochemical gradient, can dissipate the build-up of positive charge in the endosome by the accumulated H^+^. Because the affected gene was identified as a member of the *CLCN* gene family (Koch et al., 1992) the supposition that CLC-5 was also a Cl^−^ channel would have been both logical and uncontroversial. Secondly, immunohistochemical studies with CLC-5 antisera pinpointed CLC-5 to subapical endosomes in proximal tubule epithelia, colocalizing with H^+^-ATPase (Gunther et al., 1998). These ideas were supported by studies on mice lacking CLC-5, which exhibit several of the features characteristic of Dent's disease, where loss of CLC-5 was linked to defective endosomal acidification and receptor-mediated endocytosis (Piwon et al., 2000; Wang et al., 2000; Silva et al., 2003). Acidification is important because it promotes the dissociation of endocytosed protein from the receptor, megalin and cubulin (Christensen et al., 2009), and allows the recycling of the receptor back to the apical membrane for further endocytosis. It is also important for the process of endosomal maturation and enzyme activation along the endosomal-lysosomal pathway. Defective endocytosis through altered endosomal function thus explains the low molecular weight proteinuria that is the hallmark of Dent's disease and its downstream effects.

Is 2Cl^−^/H^+^ transport by CLC-5 compatible with an electrical shunt or does this transporter contribute to cellular physiology in another way? Since the exchange stoichiometry involves one H^+^ exchanged for two Cl^−^, each cycle would neutralise the charge of three H^+^ pumped in by the ATPase, but at the cost of one H^+^ transported out by CLC-5. Surely nature would have evolved a more efficient process by making use of any of the available Cl^−^-selective ion channels or does CLC-5 do something unique? In this mini-review we discuss the changing ideas of the role of CLC-5 in endosomal physiology and its pathophysiology in Dent's disease. Certainly, CLC-5 remains a major contributor to endosomal function, but studies that interrogate the precise role provide confusing and conflicting conclusions. The reader is also referred to other recent reviews in the field that explore this area and other more comprehensive aspects of intracellular CLC and organelle function (Plans et al., 2009; Wellhauser et al., 2010; Scott and Gruenberg, 2011).

## Non-transporting properties of CLC-5

Before considering the ion transport activity of CLC-5 we first ask if this is its only role or even if it is important at all. Electrophysiological studies show that depolarization to membrane potentials more positive to 0 mV are required to activate CLC-5 (Friedrich et al., 1999; Smith and Lippiat, 2010a). Surface-expressed transporters would rarely experience these potentials if at all under normal conditions and so transport-related activity at the plasma membrane would be unlikely. Furthermore, we do not understand how the potential across endosomal membranes might be regulated. Aside from this, changes in the distribution and trafficking of other plasma membrane proteins that are important to the process of endocytosis have been reported in proximal tubule cells; these include H^+^-ATPase (Moulin et al., 2003), megalin and cubulin (Christensen et al., 2003), and the NHE3 Na^+^/H^+^ exchanger (Lin et al., 2011). The trafficking and colocalization with CLC-5 may be due to protein-protein interactions in either surface or endosomal membranes, which would be lost when CLC-5 is absent or deficient. For example NHE3 has been shown to cluster with CLC-5 via the NHERF3 scaffolding protein (Hryciw et al., 2012). An alternative explanation for altered trafficking of membrane proteins could be as an effect secondary to impaired organelle acidification.

The CLC-5 intracellular domains contain a PY motif, which can interact with E3 ubiquitin ligases. Ubiquitinylation might mark CLC-5 for internalization by endocytosis, resulting in vesicle formation, or target intracellular CLC-5 directly to endosomes rather than the plasma membrane (Schwake et al., 2001; Hryciw et al., 2004). Consistent with this, co-expression of ubiquitin ligases reduced the surface density of CLC-5, whereas it was increased by mutation of the PY motif (Schwake et al., 2001; Hryciw et al., 2004). However, transgenic mice expressing CLC-5 with the same mutation that disrupted the PY motif appear normal and do not have defective endocytosis (Rickheit et al., 2010). This was a somewhat surprising finding and suggests that this manner of regulation by protein interaction and post-translational modification is not crucial for endocytosis. Ubiquitinylation of CLC-5 may promote lysosomal degradation of the protein, and so a decrease in degradation might explain the increase in CLC-5 at the cell surface through reduced recycling. Another interaction, involving the C-terminal domains of CLC-5 with KIF3B, part of the kinesin motor complex, is important for the transport of CLC-5-containing vesicles along microtubules away from the apical membrane (Reed et al., 2009). This process may also involve the actin-binding and remodeling enzyme cofilin, which also interacts with the intracellular domains of CLC-5 and regulates albumin uptake (Hryciw et al., 2003). Disease-causing mutations that alter the subcellular expression of CLC-5 (Smith et al., 2009) or disrupt C-terminal interaction domains may, therefore, affect the process of vesicular transport and contribute to the pathophysiology of Dent's disease (Reed et al., 2009). Overall, these studies suggest that irrespective of ion transport activity the presence of particular CLC-5 protein domains is likely to be important and contribute to the cellular biology. Expression of transport-deficient CLC-5 protein could be employed to investigate this further.

## Is CLC-5 active in endosomes?

The major problem in defining a physiological role for CLC-5 in its native environment is that it exerts its influence and putative ion-transporting abilities within the confines of subcellular endocytic vesicles. Unlike the study of ion channels and transporters at the cell surface, these membranes are generally inaccessible to patch clamp micropipettes and the ability to define the ionic environment. It has not been possible to determine CLC-5 activity directly and the direction of ion flux through the active transporter in either isolated endosomes or in the intact cell. Fluorescent indicators have been loaded or targeted to endosomes that report changes in luminal pH or [Cl^−^] (Gunther et al., 2003; Hara-Chikuma et al., 2005; Smith and Lippiat, 2010a). These experiments are unable to identify precisely the routes taken by H^+^ and Cl^−^ between cytoplasm and endosome, but inhibition of H^+^-ATPase (e.g., by bafilomycin-A1) or Cl^−^ channels (e.g., by NPPB) have been used to establish their relative contribution. Much of our understanding of CLC-5 function has come from overexpression studies and the properties of a minority population that resides in the plasma membrane. Electrophysiological studies show that overexpressed wild-type CLC-5 at the plasma membrane can only transport ions when depolarized to positive membrane potentials. At present, we do not know if voltage-dependent 2Cl^−^/H^+^ transport by CLC-5 is maintained in endosomal membranes or if a different behavior exists. If the biophysical properties are consistent then the cytoplasm must be more positive to an endosomal-negative potential (equivalent to depolarization) for activity to occur. In this case, electrogenic H^+^ pumping into the endosome by H^+^-ATPase would generate an endosomal positive potential which would render CLC-5 inactive.

Pharmacological tools that enable the selective and acute inhibition of CLC-5 are absent. Unlike many Cl^−^ channels and transporters, CLC-5 is insensitive to the commonly-used non-selective inhibitors such as NPPB, DIDS, and niflumic acid (Steinmeyer et al., 1995; Mo et al., 1999). These drugs have been used to provide evidence for involvement of Cl^−^ channels in receptor-mediated endocytosis or endosomal acidification, but in such studies CLC-5 must be discounted as the drug-sensitive Cl^−^ pathway and a different drug-sensitive channel or transporter protein must be present in proximal tubule endosomes (Hara-Chikuma et al., 2005).

CLC-5 function in endosomes has, therefore, been studied by comparing cellular function between cells that have and those that lack functional CLC-5. Endosomal accumulation of both H^+^ (acidification) and Cl^−^ is reduced in proximal tubule cells from CLC-5 knockout mice (Hara-Chikuma et al., 2005). Findings such as this could be explained by different underlying mechanisms: a defect in H^+^ transporting protein, a Cl^−^ conductance, or a loss of a scaffolding protein that clusters H^+^ and Cl^−^ transporters/channels to the same endosomal membrane. A reduction in endosomal acidification or [Cl^−^] could be the result of either the loss of a Cl^−^ conductance, which reduces acidification, or of defective H^+^ transport, which would reduce the amount Cl^−^ required to enter the endosome to dissipate charge.

From the studies discussed so far it can be concluded that the presence of CLC-5 is important for endocytosis and is associated with changes in endosomal pH and [Cl^−^]. What had yet to be identified, however, is at what point in the process of endocytosis is CLC-5 active and whether it is the transport of Cl^−^, H^+^, or both that is critical.

## 2Cl^−^/H^+^ exchange is critical to endosomal acidification and kidney function

**Figure 1:**
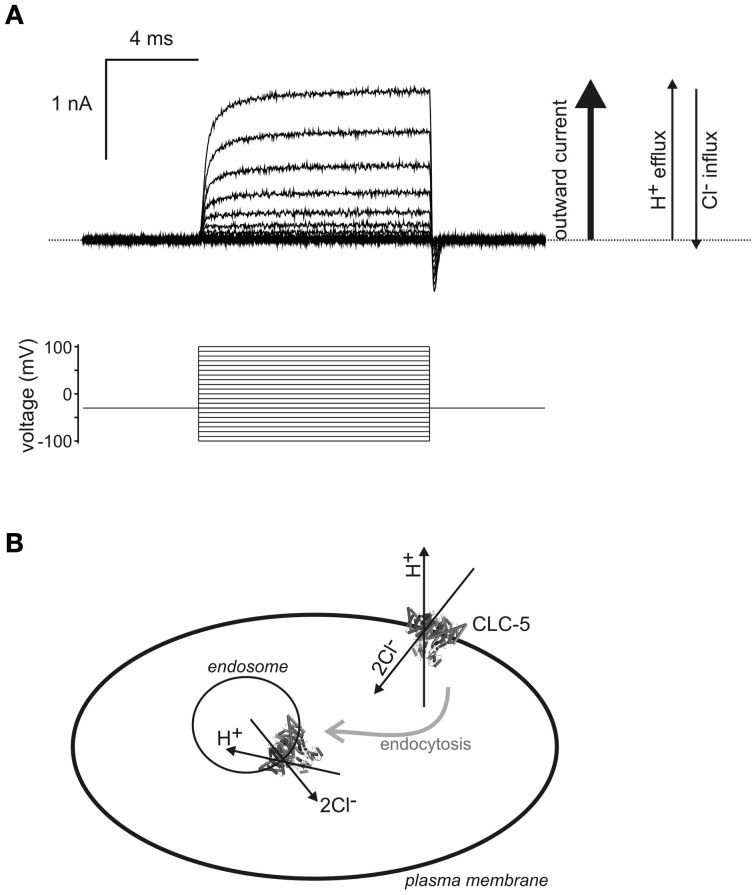
**Biophysical properties suggest transport of H^+^ into endosomes by CLC-5. (A)** A family of CLC-5 currents recorded from a transfected HEK293 cell by the whole-cell patch-clamp technique. The dotted line represents the zero current baseline. Pulses were applied from −100 to +100 mV as illustrated by the voltage protocol. Only outward currents (upward currents from the baseline), corresponding to H^+^ efflux and Cl^−^ influx, are observed with CLC-5 expressed at the cell surface, indicated by the arrows to the right of the currents. Ion flux in the opposite direction would be recorded as inward currents (downward deflection from baseline) and these were not observed under all [Cl^−^] and pH conditions tested (Smith and Lippiat, 2010a). The transient inward currents upon repolarization are gating currents and do not involve ion transport across the membrane (Smith and Lippiat, 2010b). **(B)** If this property persists in endosomal membranes then a Cl^−^ gradient, which may occur immediately upon endocytic vesicle formation, may drive the transport of H^+^ into endosomes by CLC-5. It is assumed that the arrangement of transmembrane and intracellular domains of CLC-5 are consistent between plasma and endosomal membranes, as indicated by the representation of the crystal structure of a homologous eukaryotic CLC transporter (Feng et al., 2010). Ion transport through one subunit of the dimer is indicated by the arrows and the proteins and could provides the H^+^-ATPase-independent acidification component identified by (Smith and Lippiat, 2010a). Other transporters and membrane proteins that colocalize with CLC-5 are omitted from this cartoon.

Two studies addressed the issue of ion exchange by studying the physiology of mutant CLC-5 expressed either as a transgene in mice (Novarino et al., 2010) or studied in cultured cells (Smith and Lippiat, 2010a). The E211A mutation converts CLC-5 from an outwardly rectifying 2Cl^−^/1H^+^ exchange transporter into a non-rectifying Cl^−^ conductance by uncoupling the H^+^ transport, and thereby forms a passive Cl^−^ conductance (Picollo and Pusch, 2005; Scheel et al., 2005). Mice expressing E211A CLC-5 exhibit proteinuria, similar to CLC-5 knockout mice, but ATP-dependent endosomal acidification appeared normal (Novarino et al., 2010). Novarino et al. (2010) hypothesized that endosomal Cl^−^ accumulation via CLC-5 and driven by the endosome-cytosolic pH gradient is critical for endocytosis. This would imply that Cl^−^ flowing into endosomes down its electrochemical gradient is insufficient for endosomal function and requires CLC-5 to transport additional Cl^−^ into endosomes in exchange for H^+^. This direction of ion flux through CLC-5 has yet to be observed (Figure 1) and therefore requires CLC-5 to function in novel way. Electrophysiological studies demonstrate that CLC-5 only transports ions in the opposite direction to this: only outward currents have been observed from cells overexpressing CLC-5, which correspond to transport of H^+^ from and Cl^−^ into the cytoplasm (Figure 1). One must also consider that in order to transport enough Cl^−^ ions to change endosomal [Cl^−^] even by a few millimolar, a similar amount of H^+^ must leave the endosome. This might place a considerable energetic strain on endosomal H^+^-ATPase, which is likely to have a turnover rate at least an order of magnitude lower than that of a typical CLC transporter (Jayaram et al., 2008), and the latter could readily deplete endosomal H^+^ by functioning in this way. In any case, measurements of endosomal pH reveal that the presence of CLC-5 results in a lower pH, which is inconsistent with it providing a means for H^+^ to leave endosomes (Hara-Chikuma et al., 2005; Smith and Lippiat, 2010a) and any passive Cl^−^ conductance in endosomal membranes may make it difficult to maintain high endosomal [Cl^−^].

In agreement that 2Cl^−^/H^+^ exchange was the critical process we showed, using a targeted pH indicator, that wild-type CLC-5 could enhance endosomal acidification when overexpressed in HEK293 cells, but this effect was not reproduced by expressing either the uncoupled Cl^−^-transporting mutant (E211A) nor by a transport-null mutant (E268A) (Smith and Lippiat, 2010a). Since the electrophysiological properties of CLC-5 suggested that it could only transport H^+^ from and not into the cytoplasm (Figure 1) the study inferred that CLC-5 provided a mechanism for endosomal acidification distinct from the active pumping by H^+^-ATPase. This idea was supported by identifying the bafilomycin-sensitive (H^+^-ATPase) and insensitive (CLC-5) acidification components in both the recombinant expression system and in cultured proximal tubule cells (Smith and Lippiat, 2010a). This process restricts the contribution of CLC-5 to endosomal acidification when there is sufficient endosomal [Cl^−^] and when there is an endosomal-negative transmembrane potential. This may only occur immediately upon vesicle formation with high [Cl^−^] in the internalized fluid and an interior membrane surface charge, extruding Cl^−^ into the cytoplasm during the first min (Sonawane and Verkman, 2003), which might involve 2Cl^−^/H^+^-exchange by CLC-5 in addition to any contribution from other transporters and channels. This would rapidly acidify CLC-5-containing vesicles immediately upon formation and support constitutive endocytosis by proximal tubule epithelia. The passive Cl^−^ conductance that is important for maintaining electroneutrality during H^+^-ATPase activity could be provided by the NPPB-sensitive Cl^−^ conductance (Hara-Chikuma et al., 2005) and may involve CFTR, which is found in endosomal membranes in proximal tubule epithelia (Jouret et al., 2007). Other Cl^−^-permeable channels that have detectable endosomal localization in proximal tubule cells are CLIC1 (Ulmasov et al., 2007) and AQP6 (Yasui et al., 1999a,b).

The two distinct mechanisms described here are not mutually-exclusive, and might describe CLC-5 function at different stages of the process of endocytosis (Novarino et al., 2010) when ionic gradients and maybe the voltage across the endosomal membrane change. The biophysical properties of CLC-5 may be altered by organelle-specific phospholipid content that exists in early and late endosomes (Falkenburger et al., 2010), or as a result of a yet unidentified modification.

## Summary and prospective

Defective endocytosis occurs when CLC-5 function is impaired, either in Dent's patients with *CLCN5* mutations, or in mouse models with altered or lacking *Clcn5* gene. In addition, mutant CLC-5 that can only conduct Cl^−^, and not H^+^, across endosomal membranes cannot support endosomal function (Novarino et al., 2010; Smith and Lippiat, 2010a). The importance of H^+^ transport must, therefore, be the key to understanding CLC-5 and the idea that it provides a means to provide Cl^−^ to electrically shunt endosomal H^+^ accumulation is redundant. Questions remain as to when during endocytosis and endosomal acidification CLC-5 is active and the direction of Cl^−^ and H^+^ fluxes. Any method that enables the manipulation of endosomal membrane potential and ionic content is likely to help dissect out these processes. As discussed here, the importance of coupled transport by CLC-5 and the redundancy of the PY motif have been determined by studying mutant transporters. Identification of other mutations that alter just one process, e.g., voltage-dependence or exchange stoichiometry, and studying their effects at the cellular and whole-animal levels may prove to be useful in the future.

Knowledge of the full endosomal membrane proteome should also provide insights into endosomal ionic regulation since transporters and channels often work in conjunction to provide constitutive activity. It is unlikely that CLC-5, H^+^-ATPase, and NHE3 exist in isolation as ion-transporting proteins in endosomal membranes in the proximal tubule. In addition to anion channels, evidence for the endosomal function of cation-selective channels from the mucolipin (TRPML) and two pore channel (TPC) families is emerging (Grimm et al., 2012). Release of calcium from the newly-formed endocytic vesicle through mucolipin-3 (TRPML3) channels is required for endosomal acidification in retinal epithelial cells (Lelouvier and Puertollano, 2011). TPC proteins expressed in endosomal and lysosomal membranes give rise to a largely Na^+^-selective channel which, upon activation by phosphatidylinositol 3,5-bisphosphate, allows Na^+^ to leave the lumen (Wang et al., 2012). Release of cations from endosomes might actually depolarize the endosomal membrane (Wang et al., 2012), which would favor and maintain CLC-5 activation. Further investigation is required in order to establish whether either cation channel type is expressed and colocalized with CLC-5 in proximal tubule cells.

Studying ion homeostasis and transport in organelles is a challenge and requires development of selective and targeted fluorescent indicators or the means to interrogate ion channel and transporter function *in situ*. Imaging and optogenetics may thus provide tools to enable experiments on intact cells and organelles. Organelle electrophysiology where endosomal membrane potential and ionic content could be controlled is technically demanding and is limited by their small size, although studies on artificially-enlarged endosomes or endolysosomes have been successfully implemented (Saito et al., 2007; Wang et al., 2012). CLC-5 is critical for endocytosis and endosomal function in proximal tubule epithelia. Until we are able to resolve its behavior in more detail, we must suffice ourselves with the conclusion that it is because of a unique property not shared with any other class of Cl^−^-transporting protein found in the kidney, 2Cl^−^/H^+^ exchange.

### Conflict of interest statement

The authors declare that the research was conducted in the absence of any commercial or financial relationships that could be construed as a potential conflict of interest.
